# Commentary: Transorbital approach to the cavernous sinus: an anatomical study of the related cranial nerves

**DOI:** 10.3389/fnana.2024.1428516

**Published:** 2024-07-02

**Authors:** Sergio Corvino

**Affiliations:** Department of Neurosciences and Reproductive and Odontostomatological Sciences, Division of Neurosurgery, University of Naples “Federico II”, Naples, Italy

**Keywords:** endoscopic transorbital approach, cavernous sinus, anatomical triangles, skull base, endoscopic endonasal approach (EEA)

## Introduction

I read with interest the article titled “*Transorbital approach to the cavernous sinus: an anatomical study of the related cranial nerves”* by Mosteiro et al. (2024), in which the authors provide an “*anatomical description of the cavernous sinus, the course of III, IV, VI, and V cranial nerves, and C3–C7 segments of the internal carotid artery from the ventrolateral endoscopic transorbital perspective”* through anatomical dissections on cadaveric head specimens.

For a long time, the cavernous sinus (CS) has been considered the “anatomical jewel box” and surgical “no man's land” due to the vital and highly functional neurovascular structures hosted within its boundaries and the high risks associated with surgery. Nevertheless, since the first pioneering study of Parkinson (1965), this deep-seated venous space in the central skull base has attracted great interest among neurosurgeons, who have investigated additional surgical routes from different access perspectives, including, more recently, the endoscopic transorbital approach (Dallan et al., 2017; Jung et al., 2022; Corvino et al., 2023d; Evins et al., 2024).

In fact, since the introduction of the TONES (Trans-Orbital Neuro-Endoscopic Surgery) concept (Moe et al., 2010) in 2010, the surgical indications of endoscopic transorbital approaches for neurosurgical pathologies are rapidly increasing thanks to its peculiar advantages (Corvino et al., 2023a). Transorbital approaches allow to address lesions involving the paramedian regions of the anterior and middle skull base, as standard or extended, single or combined multiportal approaches (Di Somma et al., 2022; Corvino et al., 2023b, 2024a; de Notaris et al., 2023).

## Discussion

Previously, our group have investigated the surgical anatomy of the CS via multiple surgical corridors, including fronto-temporal-orbito-zygomatic (FTOZ), endoscopic endonasal and endoscopic transorbital approaches. Each of the well-known anatomical triangle and the relative anatomic landmarks for each approach were separately analyzed. In addition, we attempted to identify the safe entry zones to this dural envelope and provide indications on the approach selection (Corvino et al., 2023c,d).

I would like to commend the authors (Mosteiro et al., 2024) for going beyond this previous research by exploring the intradural and cisternal spaces and by following the course of the cranial nerves crossing the cavernous sinus, to further expand the potential application of the endoscopic transorbital approach.

Concerning the cavernous sinus surgery, it is a challenge for the highly vulnerable neuroanatomical structures contained within. FTOZ approach allows access to the cavernous sinus from above via clinoidal and oculomotor triangles, and laterally via supra and infra-trochlear triangles. The extended endoscopic endonasal approach allows access to the posterosuperior and anterior compartments of the CS from its medial wall. To enter the posterior compartment, an opening of the sellar dura and the sacrifice of the inferior hypophyseal artery are required, while access to the anteroinferior compartment is facilitated by the transpterygoid approach. The endoscopic transorbital approach exposes the lateral wall and part of the posterior wall (Corvino et al., 2023d).

The selection of the approach mainly depends on the relationship of the lesion with the cranial nerves and on the physiological/pathological course of the internal carotid artery. In general, lesions located medial to the cranial nerves are more suitable for endonasal and/or transcranial approaches; lesions located lateral to the cranial nerves are more suitable for transcranial and transorbital corridors.

A cadaver-based micro-neurosurgical laboratory represents an optimal environment where developing, improving and refining the microsurgical anatomy knowledge and surgical skills through fine dissections and simulating neurosurgical operative procedures. Over the last years many alternative or complementary methods to the cadaveric dissections have been proposed for anatomy learning, including digital teaching, 3D printing, virtual augmented reality, surgical simulation and photogrammetry (Corvino et al., 2024b). In addition, anatomical research based on cadaveric dissection has led to the continuous development of new surgical corridors and to even more tailored surgical procedures, accordingly, resulting in decreased mortality and morbidity rates. The ongoing advancements in diagnostic tools, as well as in surgical techniques and technology, continue to push the boundaries of what is possible in cavernous sinus surgery, further enhancing the safety and efficacy of surgery. Future research and innovation will likely yield even more refined approaches, ultimately improving patient outcomes.

## Author contributions

SC: Conceptualization, Data curation, Validation, Writing – original draft.
